# Application of an Instrumental and Computational Approach for Improving the Vibration Behavior of Structural Panels Using a Lightweight Multilayer Composite

**DOI:** 10.3390/s140304960

**Published:** 2014-03-11

**Authors:** Alberto Sánchez, Manuel García, Miguel Angel Sebastián, Ana María Camacho

**Affiliations:** 1 Department of Materials Science and Metallurgical Engineering, Graphic Expression in Engineering, Cartographic Engineering, Geodesy and Photogrammetry, Mechanical Engineering and Manufacturing Engineering, Universidad de Valladolid, P° del Cauce 59, Valladolid 47011, Spain; 2 Department of Construction and Manufacturing Engineering, Universidad Nacional de Educación a Distancia (UNED), C/Juan del Rosal 12, Madrid 28040, Spain; E-Mails: mggarcia@ind.uned.es (M.G.); msebastian@ind.uned.es (M.A.S.); amcamacho@ind.uned.es (A.M.C.)

**Keywords:** vibration sensors, ODS instrumental test, FEM, improvement, composite

## Abstract

This work presents a hybrid (experimental-computational) application for improving the vibration behavior of structural components using a lightweight multilayer composite. The vibration behavior of a flat steel plate has been improved by the gluing of a lightweight composite formed by a core of polyurethane foam and two paper mats placed on its faces. This composite enables the natural frequencies to be increased and the modal density of the plate to be reduced, moving about the natural frequencies of the plate out of excitation range, thereby improving the vibration behavior of the plate. A specific experimental model for measuring the Operating Deflection Shape (ODS) has been developed, which enables an evaluation of the goodness of the natural frequencies obtained with the computational model simulated by the finite element method (FEM). The model of composite + flat steel plate determined by FEM was used to conduct parametric study, and the most influential factors for 1st, 2nd and 3rd mode were identified using a multifactor analysis of variance (Multifactor-ANOVA). The presented results can be easily particularized for other cases, as it may be used in cycles of continuous improvement as well as in the product development at the material, piece, and complete-system levels.

## 1. Introduction

The vibration behavior of a structural panel is an important factor in the operation of a machine or structure. A design that does not consider the vibration behavior can cause malfunctioning, noise, discomfort, and/or injuries to the user and even failure of the ensemble. Experimental modal analysis (EMA) is a technique that enables the determination of the resonances of a machine, identification of its modal parameters, and prediction of its vibration behavior [1]. In many cases, it is necessary to know the vibration mode of a structure or a machine under its own operation mode, in which an EMA can be limited—for example, by the impossibility of measuring the excitation force [2]. The Operating Deflection Shape (ODS) can be defined as the deformation of a structure at a determined frequency. The ODS measurement provides information regarding the operation conditions; it enables the determination of how and to what magnitude the machine has moved, whether resonance of the machine is excited, and the type of modal deformation involved. Mode Shape and ODS are related to one other (see Table 1). In general, all experimental modal parameters can be obtained from measured ODSs. At or near one resonance peak, the ODS is dominated by a mode. Thus ODS is approximately equal to the mode shape [3]

ODS analysis has already been successfully used for detecting damage in beams, bridges, turbines, machines, and various types of structures [4–11], noise control [12], evaluating the vibration behavior of machine tools [13], bladed disks [14], a machine press [15], musical instruments [16,17] and grass trimmer systems [18–20], and determining the optimum location of the sensors in sanders and polishing machines to accurately evaluate the risk of hand-arm vibration exposure [21].

In the present work, a hybrid (instrumental-computational) approximation based on a finite element computational model (FEM) is proposed, along with an instrumental technique that applies ODS analysis. This approximation allows for experimentally estimating the modal response of the system (mainly the natural frequencies) under determined test conditions, thereby validating the resonance frequencies obtained by the FEM simulation model. To demonstrate the validity of this approximation, the improvement of the vibration behavior of a thin steel sheet is proposed. Thin steel sheets are of great interest to the design of engineering structures such as car bodies, ships, aerospace structures and building structures [22].

Generally, an undesired vibration level is corrected by decreasing the modal density and displacing the natural frequencies, taking them out of the operation range, increasing the damping and/or the mass, or increasing the rigidity of the ensemble. In the presented practical application, the goal is to reduce the modal density to the interval of 0 to 150 Hz and to increase the first resonance frequency to values above 70 Hz. To increase the resonance frequencies, rigid elements can be added to the vibrating surface, or the geometry can be modified by moldings or curvature changes. Different studies present the results of these strategies, which, although providing a good solution to the vibration problem, can involve increased weight and cost and may even cause manufacturing problems or lead to important modifications in the design [23–34]. Another route to solving this type of problem is the so-called “active control” [35–39]. In sectors such as the automotive sector, weight is a strategic factor that requires the use of lightweight materials capable of maintaining or improving the current solutions employed [40–42]. To improve the vibration behavior of the proposed steel sheet plate, a lightweight multilayer composite composed of a polyurethane foam core and two paper mats has been used. This composite is applicable, among other uses, in automotive components. A correct design of this composite raises the damping of the thin steel sheet and the resonance frequencies. The composite moves about the resonance frequencies out of the range where the thin steel sheet could produce noises or failures, thereby improving the vibration behavior of the plate.

The present study differs from previous ODS studies [22,43] in that all the perimeter of the plate is now excited by a electrodynamic shaker with pink noise instead of exciting it at a single point and frequency at a time. The main interest is now not to measure the eigenmodes, but the ODSs and the resonance frequencies. It also differs from investigations where the vibrational behaviors of thin steel sheets have been studied in that this work presents a composite computational model simulated by the finite element method (FEM), this model was used to conduct parametric study, and the most influential factors for 1st, 2nd and 3rd mode were identified using an ANOVA multifactor analysis. This studied composite formed by a core of polyurethane foam differs from investigations where acoustical absorption is the main function of this type of composite [44]. The experimental tests have been performed with low-complexity instrumentation. Studies of modal parameters involving OSD measurements have considered other types of structures and materials [4–21], or boundary conditions [45–48].

## 2. Experimental Section

This section describes on the one hand the methodological approach and on the other hand the tested samples, the experimental test method, and the finite element calculations performed for the practical case.

### 2.1. Methodological Approach

**Figure 1.**
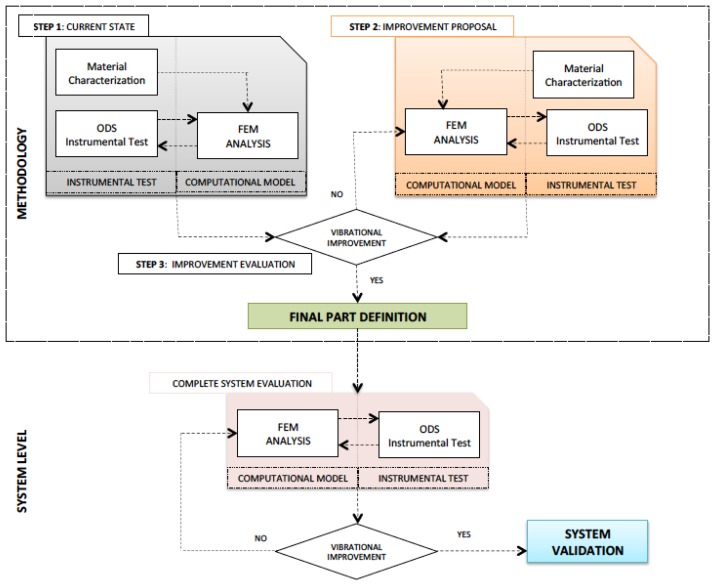
Hybrid approach to improve vibrational behavior of structural panel.

The applied methodology (Figure 1) is developed with both an experimental and a computational approach, ultimately providing the definition of an ensemble that improves the reference vibration state.

First (step 1), the material of the system to be improved and its vibration behavior is characterized at a test piece level. The material characterization will be used as input data in the computational model. The results of the experimental test will serve to evaluate the validity of the computational model simulated by finite elements (FEM). In this manner, two objectives are reached. On the one hand, the hybrid experimental-computational work methodology (ODS-FEM) is validated; on the other hand, there is a reference for the vibration behavior of the initial system (see step 1: current state, Figure 1).

With this reference, different proposals are given for the improvement of its vibration behavior. With the chosen proposal/s, a computational model is generated, and the experimental tests are performed in the same manner as in the previous step (see step 2: improvement proposal in Figure 1). With the comparison of the results obtained in steps 1 and 2, the improvement of the vibration behavior is evaluated (step 3). Consequently, the parameters with which the piece will be made (composition, thickness, density, *etc.*) are determined. This piece may be adjusted and/or validated at a complete system level using the results obtained when applying this methodology (materials, experimental test definition (ODS) and development of the computational model (FEM)) (see Figure 1).

### 2.2. Samples

**Figure 2.**
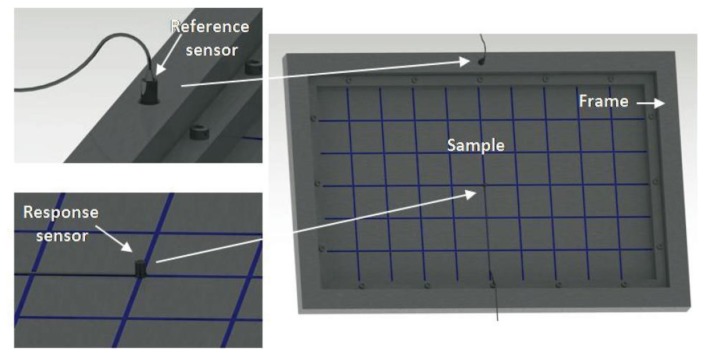
ODS measurements: measurement points, sensors and sample location.

In the presented practical application, the aim is to improve the vibration behavior of a steel sheet with a thickness of 0.8 mm, a density of 7700 Kg/m^3^, and dimensions of 540 × 340 mm^2^. The plate is fixed in a frame by 16 bolts to a rigid cabin (Figure 2). The tests and simulations performed on this plate are used as a reference to improve the vibration behavior of the plate.

To improve the vibration behavior of this plate, a multilayer composite with a thickness of 14 mm, a density of 69.8 Kg/m^3^, and dimensions of 540 × 340 mm^2^ is glued to the plate. This element is composed of an open-cell polyurethane foam core and two paper mats on its extremes. To glue the plate to the composite, five strips of a polyurethane adhesive commonly used in the manufacturing of vehicles have been used. The plate and composite assembly is fixed in a frame to a rigid cabin under the same conditions as those of the test conducted with the previously described plate. This composite enables the natural frequencies to be increased and the modal density of the plate to be reduced, moving about the natural frequencies of the plate out of excitation range, thereby improving the vibration behavior of the plate.

### 2.3. Experimental Test: ODS Measurements

Instead of performing an EMA, an ODS analysis has been used to estimate the modal characteristics of the tested samples. The modal deformations and ODS are different, but the relationship between both can be used to obtain the modal characteristics (resonance frequencies, damping factors, and modal deformations) by measuring the ODS without the necessity of knowing the excitation force [49–52].

Out of all the existing techniques used to obtain the ODS, transmissibility measurements can be the easiest to perform due to their similarity with the Frequency Response Function (FRF). This advantage is limited by the fact that it is generally not possible to identify modal parameters with transmissibility measurements [3,52–55].

To be able to apply transmissibility measurements to estimating modal parameters with some guarantee of success, the transmissibility coherence must be high [51], and the coupling between modes must be studied. Identifying the modal parameters by the ODS is more complex in highly damped systems [56]. By knowing the location of the excitation force and restricting the movement of the system in that location, the modal parameters have been successfully identified and even correlated using FEM computational models [45–48].

A rigid cabin was used to perform the ODS experimental measurement, in which the samples were fixed with the aid of a rigid frame. Using 16 bolts, the sample was jointly fixed to the frame and to the structure that transmits the excitation force. The movement of the sample to be tested in the frame was completely restricted (Figure 2). The force was applied to the frame by an electrodynamic shaker along the vertical direction. A central point of the frame is used as a reference signal to perform the transmissibility measurements. The frame point has a flat response spectrum in the range of the frequencies of interest, which is extremely similar to the introduced force. This procedure minimizes errors in estimating the modal parameters. Under these conditions (excitation degrees of freedom are constrained), the peaks of the reference-response transference functions correspond to the system resonances, and given the light damping of the system (mainly for the naked steel sheet plate), the peak values of the imaginary part of the transference function at each point can be used to represent the ODS (approximately equal to the modal deformation), as is performed if force-acceleration transference functions would have been conducted (but without force measurement) [48,57].

Transmissibility measurements were performed with two acceleration signals at 45 points of each test sample, using as a reference the central spot of the frame in which the plate is screwed to the rigid cabin (Figure 2). Work began by measuring the naked metal sheet plate; afterwards, the low mass composite was glued to the plate (metal sheet plus the lightweight multilayer composite). Five polyurethane adhesive strips were used to glue the composite to the plate.

The force was applied in the vertical direction (perpendicular to the test samples) by an electrodynamic shaker (excitation signal: Pink Noise); the measured magnitude was the acceleration in the force direction. Table 2 presents the configuration of the measurement equipment.

The instruments used for the experimental ODS measurement were as follows: Dual-Channel Signal Analyzer Brüel & Kjær (B&K, Nærum, Denmark) Type 2032, Reference Signal Sensor: Piezoelectric Accelerometer B&K 4374, Response Signal Sensor: Piezoelectric Accelerometer B&K 4384, Power Supply B&K ES 5001, Signal Conditioners B&K 2635, Electrodynamic Shaker B&K 4809, and Accelerometer Calibrator B&K 4294.

### 2.4. Computational Study: FEM Model and Parametric Analysis

**Figure 3.**
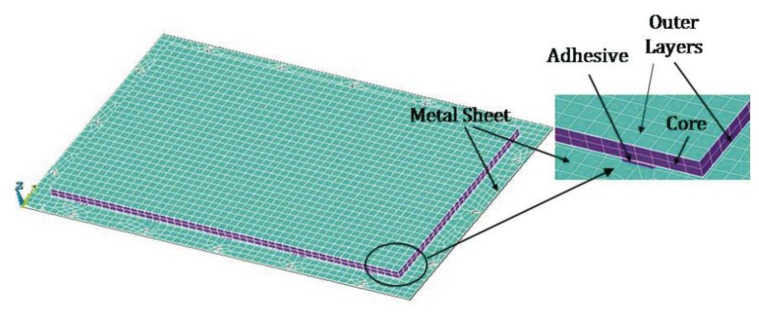
Metal Sheet and Multilayer Composite: 3D FEM.

The finite element computational model was performed using the software ANSYS^®^. The procedure began by generating the model of the naked steel plate to enable the results obtained from the simulation to be correlated with the experimental data and thereby yield a simulation reference model. Two models of finite elements were used for the naked plate: a first model in which the mounting holes of the plate were not taken into account, and a second model in which these holes were considered. The type of element used was SHELL63, which is an element developed for low-thickness plates operating under bending [58]. The calculations were run, varying the number of elements in the simulation model, the solution method (Block-Lanczos and Subspace) for the modal analysis, and the boundary conditions to observe their influence on the resonance frequencies obtained as a solution in the modal analysis simulated by the FEM. The FEM model for the metallic plate + composite is presented in Figure 3. The elements used in the composite were SOLID 45 for the core and SHELL 63 for the external layers. The adhesive was modeled with SOLID 45.

The mechanical properties (including the damping properties) can be obtained by different experimental tests [59–62]. In our case, the Young's modulus and Poisson's ratio of the steel sheet and the composite used in the simulation models were provided by the material suppliers and scientist references [63,64].

The obtained results enable the development of a simulation model for the composite-metallic plate system that was used in a parametric analysis of the system to examine the influence of the Polyurethane Core Thickness, Polyurethane Core Type, Outer Layers and Adhesive Type (see Table 3) The results of the simulations were subjected to an analysis of variance (ANOVA) to determine whether there were significant differences at the 95% level of confidence; the analysis was conducted using the Statgraphics software [65]. The Fischer-Snedecor test uses the *F*-ratio and the *P*-value: a *P*-value greater than 0.05 implies that there are no significant differences between the means of the two sets of data [66]. This analysis was performed for the simulation results of the first mode, second mode and third mode to determine which of the factors—thickness Foam (A), Foam Type (B), Outer Layers (C), and Adhesive Type (D)—and their interactions (A-B, *A*-*C*, A-D, B-C, B-D and *C*-*D*) were significant in the results.

## 3. Results and Discussion

As a practical application of the methodology, it is proposed that the modal density of the steel sheet plate described in Section 2.2 be decreased to the frequency range of 0 to 150 Hz, also displacing the first mode of the plate to a frequency higher than 70 Hz. It is intended to achieve these goals without changes in the plate geometry and without a significant increase in the plate mass. For this purpose, a composite is adhered to the plate, as described in Section 2.2. The results obtained for the situation to be improved (Naked Metal Sheet) and the improvement proposal (metal sheet plus the lightweight multilayer composite) are described in this section.

### 3.1. Step 1: Current State (Naked Metal Sheet)

#### 3.1.1. ODS Experimental Measurements

**Figure 4.**
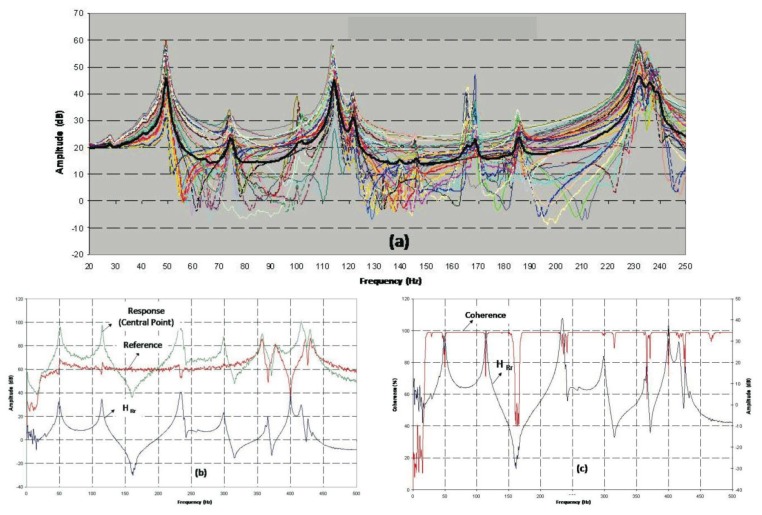
(**a**) Naked Metal Sheet Transmissibility (**b**) Response and Reference Autospectrum (**c**) Coherence and Transmissibility (Central point and Reference).

Figure 4a displays the 45 transmissibility functions following the experimental test described in Section 2.3. The phase value and the spectrum of each accelerometer have been collected in each measurement (Figure 4b), in addition to the modulus (real part) and the imaginary part of the transmissibility function and the coherence in each measurement (Figure 4c).

In Figure 4b, it can be observed that the spectrum collected as the reference is practically flat above the range of frequencies of interest (0–300 Hz). Therefore, only the transmissibility function peaks below 300 Hz have been taken into account. It is observed that where a peak appears in the transmissibility function, a change in the phase and a slight loss of coherence can be appreciated (Figure 4c). To avoid losing information on certain resonance frequencies, a fictitious curve “H-Sum” is generated, which is obtained by averaging all the measured transmissibility functions (black curve, Figure 4a). Observing this curve, we can detect the peaks: 50.6; 73.8; 114; 123; 148; 168; 186; 233 and 238 Hz, considered as resonances, for the naked metallic plate (for the objectives marked, we are interested in frequencies below 150 Hz).

#### 3.1.2. FEM: Modal Analysis

The ANSYS**^®^** commercial software was used in the simulated modal analysis of the naked steel plate, as described in Section 2.4. The results of the different simulation models for the naked metallic plate reveal the following:

- A thinner meshing of the model generates results slightly higher in frequency, although when reaching a limit, the results do not vary considerably.
- The Block-Lanczos method generates a solution with higher frequencies than the Subspace method (mainly for frequencies above 200 Hz).

Including the drillings in the model makes the solution frequency closer to the experimental results (ODS). In general, adding boundary-condition restrictions to the same model increases the frequencies. The FEM model that most closely approximates the experimental results has the edges of the drills recessed and restrictions of xyz turning and z displacement in the plate-rigid frame contact zone. These boundary conditions are the most similar to those present in the experimental test. Performing a comparison with this method and the measured ODS, and always using the experimental ODS as a reference, resonance frequencies for the naked metallic plate are obtained; these values are: 50.8; 73.7; 113.2; 127.5; 148.8; 168.3; 185.6; 243.4 and 238.9 Hz

#### 3.1.3. ODS *vs.* FEM Results

**Figure 5.**
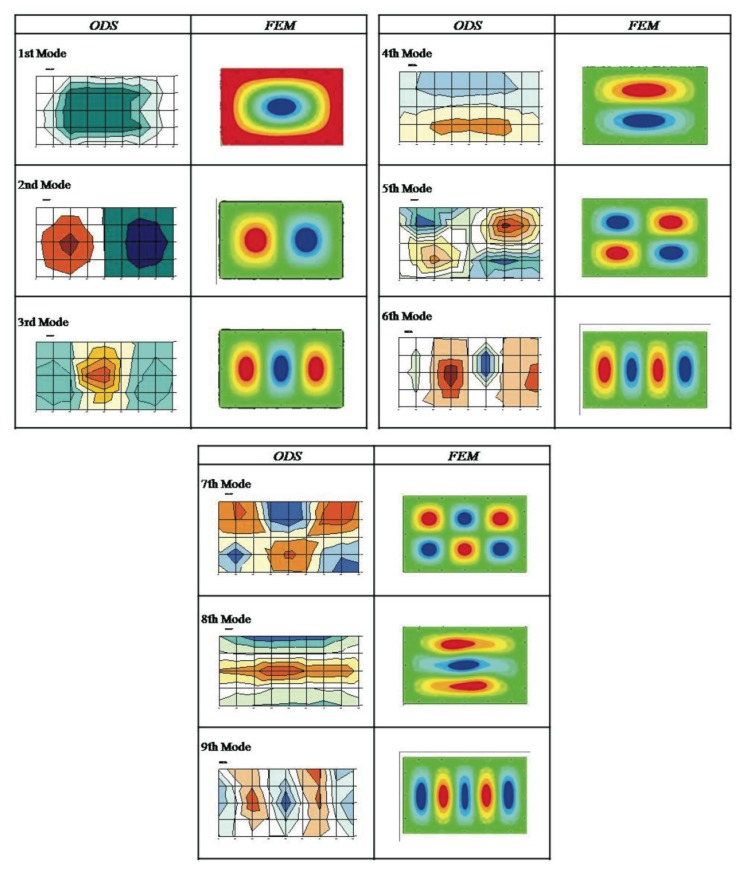
Naked Metal Sheet: ODS and Mode Shape (FEM).

The following section presents the correlation between the natural frequencies obtained with the modal analysis by finite elements and the experimentally measured ODS. Table 4 lists the natural frequencies with equivalent deformations for the FEM simulation model and the experimental ODS measurements. Figure 5 presents a comparison of the modal deformations and the experimentally obtained ODS. Because the eigenvalues of the experimental modes are not available (the ODSs have been measured), comparison criteria (Modal Assurance Criterion—MAC) of the numerical and experimental modal deformations will not be established. Instead, the modal deformations are compared without establishing a comparison value by observing their shape.

### 3.2. Step 2: Improvement Proposal (Metal Sheet Plus Lightweight Multilayer Composite)

It is intended to achieve the goals without changes in the plate geometry and without a significant increase in the plate mass. For this purpose, a composite is adhered to the plate, as described in Section 2.2.

#### 3.2.1. ODS Experimental Measurements

**Figure 6.**
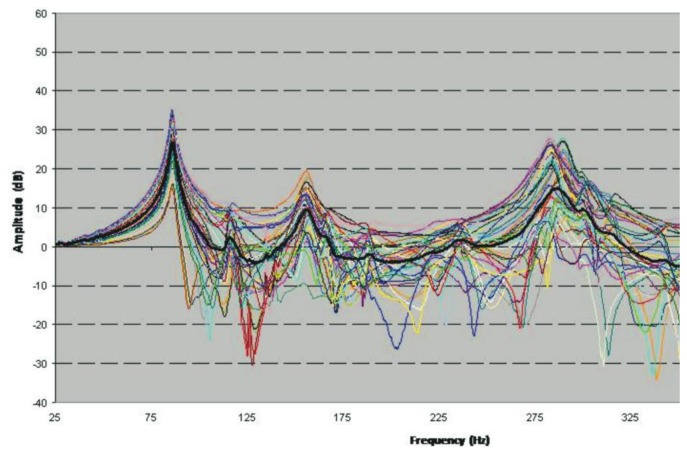
Transmissibility Naked Metal Sheet plus Lightweight Multilayer Composite.

Following the same procedure as that described for the galvanized steel sheet plate, ODS measurements were performed for the composite glued to the steel sheet plate. Forty-five transmissibility measurements were performed, generating a fictitious cure “H-Sum (black color in Figure 6).

Observing this curve, we can detect the peaks: 85.6; 118.1; 156.9; 166.3; 189.4; 221.9; 238.1; 288.1 and 301.3, considered as resonances (for the naked metallic plate plus lightweight multilayer composite (for the objectives marked, we are interested in frequencies below 150 Hz).

#### 3.2.2. FEM: Modal Analysis

The model described in Section 2.4 was used in the modal analysis calculation for the metallic plate plus composite using the same boundary conditions and resolution method as in the simulation of the naked metallic plate. The natural frequencies for the FEM simulation model are 85.9; 120.0; 165.9; 180.5; 205.0; 220.4; 243.3; 285.1 and 301.6.

#### 3.2.3. ODS vs. FEM Results

**Figure 7.**
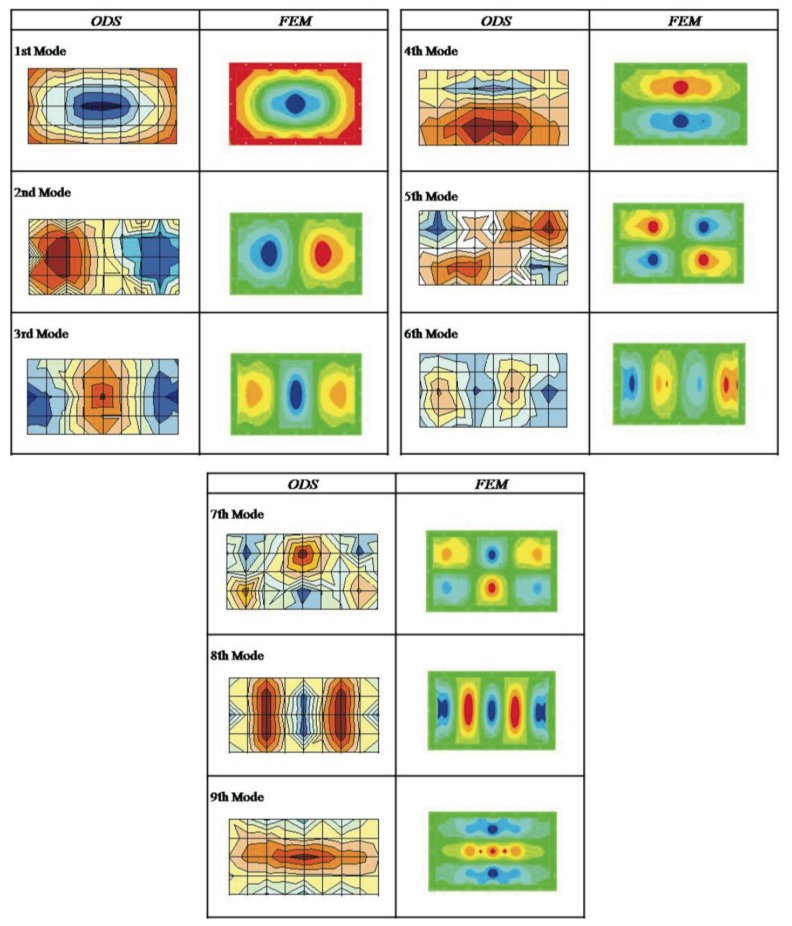
Naked Metal Sheet plus Lightweight Multilayer Composite: ODS (**left**) and Mode Shape (FEM) (**right**).

Table 5 lists the natural frequencies with equivalent deformations for the FEM simulation model and the experimental ODS measurements for the metallic plate plus composite. Figure 7 presents a comparison of the modal deformations and the experimentally obtained ODS for the metallic plate plus composite. The modal deformations are compared without establishing a comparison value by observing their shape.

### 3.3. Step 3: Improvement Evaluation

**Figure 8.**
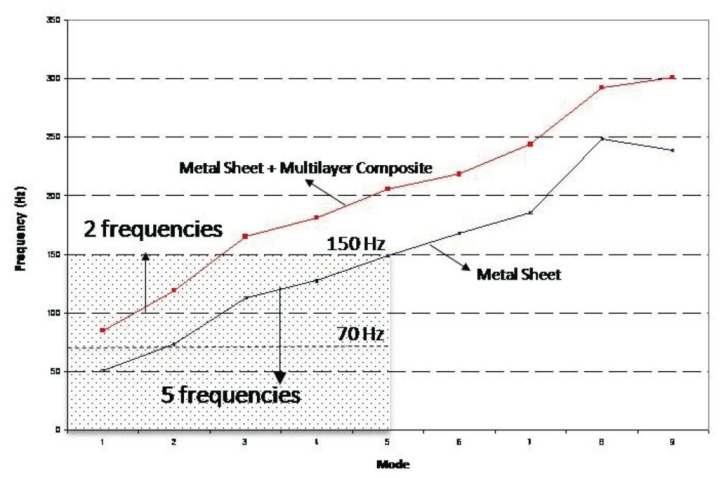
Modes: Naked Metal Sheet *vs.* Metal Sheet plus Lightweight Multilayer Composite.

Adding the composite stiffens the naked plate, causing the natural frequencies of the naked metallic plate to be displaced toward higher values (see Figure 8) and the number of resonance frequencies in the range of interest to be reduced. In addition, the vibration level decreases, thereby increasing the damping of the system (as can be observed in the peaks of Figures 4a and 6). The first resonance frequency for the ensemble is placed at a value higher than 70 Hz (85.6 Hz), going from five natural frequencies (for the naked metallic plate) to two frequencies in the range from 0 to 150 Hz (shaded area in Figure 8).

**Figure 9.**
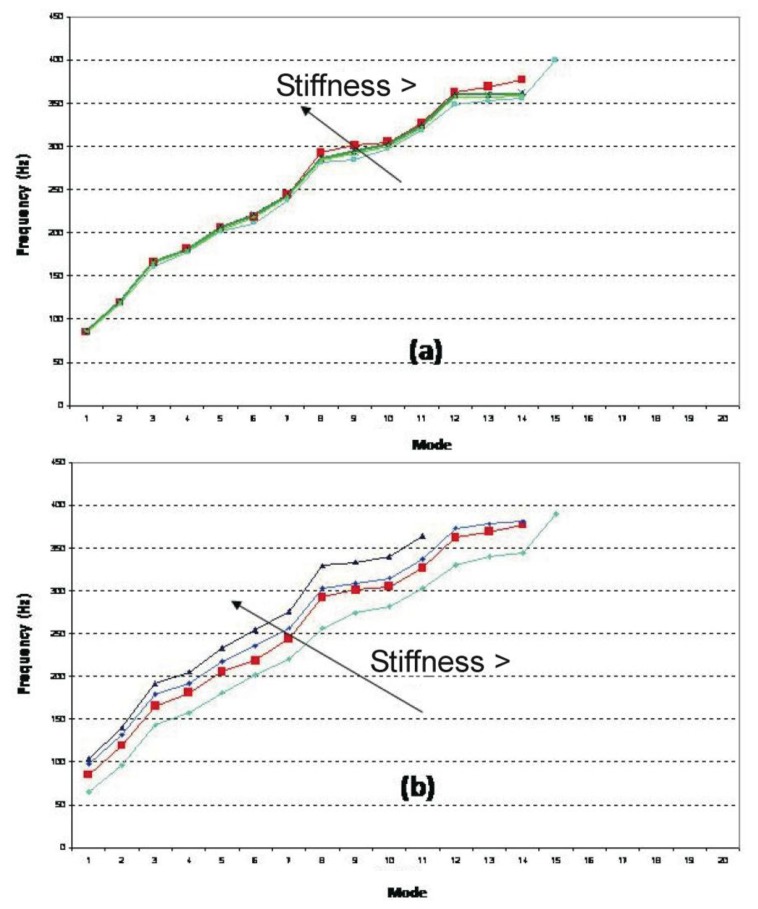
(**a**) Metal Sheet plus Lightweight Multilayer Composite: Adhesive Stiffness Analysis; (**b**) Metal Sheet plus Lightweight Multilayer Composite: Composite Stiffness Analysis.

The obtained results enable the development of a simulation model for the composite-metallic plate system that was used in a parametric analysis of the system to examine the influence of the adhesive (Figure 9a) and composite (Figure 9b) rigidity. The influence of the adhesive rigidity is extremely small, while that of the composite is more important. An increase in the composite rigidity raises the natural frequencies and decreases the modal density in the established range of interest. The red curve in Figure 9b corresponds to the material used in the experimental analysis, representing a good solution for the posed problem.

**Figure 10.**
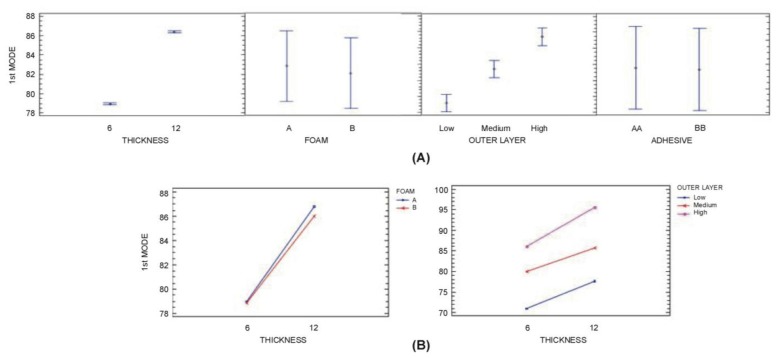
(**a**) 1st Mode (95% confidence interval (**b**) 1st Mode: A × B and A × C interactions (95% confidence interval).

The ANOVA analysis (Table 6) shows Thickness (A), Outer Layers (C), Thickness - Outer Layers (A-C) and Thickness—Foam (A-B) interactions are significant in the determination of 1st Mode (*P*-value is greater than 0.05). However, the degree of influence is much higher for A, followed by the C, A-C and A-B interaction, where the first two account for 98%. Higher thickness of polyurethane core and higher stiffness of Outer Layers increase 1st Mode (Figure 10).

**Figure 11.**
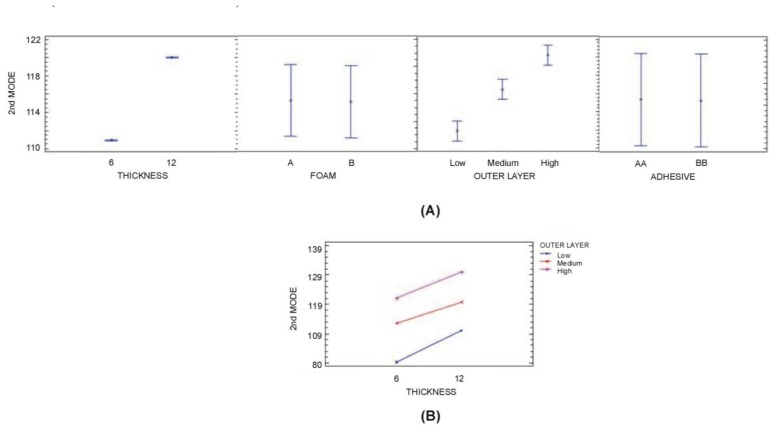
(**a**) 2nd Mode (95% confidence interval (**b**) 2nd Mode: A × C interaction (95% confidence interval).

**Figure 12.**
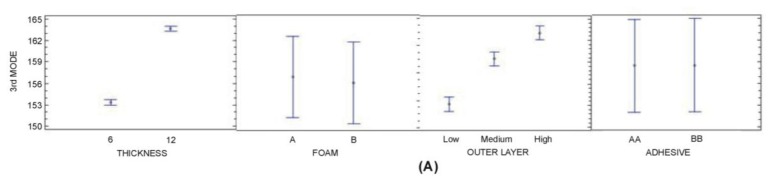

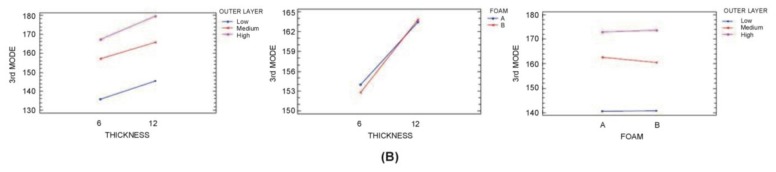
(**a**) 3rd Mode (95% confidence interval (**b**) 3rd Mode: A×B, A×C and B×C interactions (95% confidence interval).

In the analysis of 2nd and 3rd Mode (see Tables 7 and 8), it was found that factors A and C and interactions A-C are significant at a 95.0% level of confidence (in addition B-C, A-B interactions for the 3th Mode). It was also observed that nearly 99% of the variance is attributable to A and C. In general terms, higher thickness of polyurethane core and higher stiffness of Outer Layers increase 2nd and 3rd Mode (Figures 11 and 12). It can be observed that the three variables are the same variables that have the most influence on the 1st Mode.

Finally, it should be noted that adhesive type has a negligible influence on 1st, 2nd and 3rd Mode. Although the model of composite + flat steel plate determined by FEM suits the aim of this investigation, in order to study a more complicated system using the presented approach, an accurate modelling of Metal Sheet and Multilayer Composite as shown in Figure 3 would be computationally quite expensive due to the small thicknesses and necessity for a great number of elements. A sandwich modelling approaches more suitable to an industrial environment focus on equivalent shells or homogeneous three-dimensional element with isotropic properties will be viable [44]

## 4. Conclusions

The proposed methodological approach allows for obtaining the resonance frequencies of the test sample by measuring the transmissibility functions without knowing the excitation force. To relate an ODS to the resonance frequency of the test sample with a modal deformation at that frequency, it is necessary to determine the location of the force and to restrict the movement of the system to the rigid frame where the force is applied. To achieve this objective, the sample must be strongly attached to a rigid frame screwed to a rigid cabin, applying the force to the frame periphery. For the ODS measurement, it is required that all the measurement points are collected simultaneously and that the mass of the sensors does not influence the measurement. In the discussed case, the force is stationary, which allows measurements to be performed point by point, and a specific low mass sensor for the modal analysis was used. The experimental tests have been performed with low-complexity instrumentation, and these tests can be generalized as necessary. Furthermore, a computational model is generated for obtaining the reference parameters to be improved and for developing better solution proposals. Thus, it was possible to correlate this model with the experimentally measured ODS, which in turn facilitated the tuning of a solution by only performing the necessary experimental measurements.

The practical application yielded an extremely small frequency difference between the resonance frequency obtained by the computational modal analysis (FEM) and that obtained by the ODS measurement (less than 9% for the steel plate plus multilayer composite, and 4% for the naked steel plate). Thus, it is concluded that the experimental test design enables the identification of the natural frequencies of the system, overcoming the difficulties encountered when performing this identification with transmissibility measurements.

In addition, the utilized composite helped to overcome the problem, increasing the first resonance of the naked plate by almost 70% and reducing the modal density (in the range of 0–150 Hz) by 40%. The computational model enables a parametric analysis of the variables than can affect the vibration behavior of the assembly (rigidity, weight, geometry, *etc.*), and the model of composite + flat steel plate determined by FEM was used to conduct parametric study, and the most influential factors for 1st, 2nd and 3rd mode were identified using an ANOVA multifactor analysis. Influence of thickness of composite core and Outer Layers is significant. More research on the influence of polyurethane core will be conducted. These results could be extended not only to cycles of continuous improvement but also to the stages of conception, development, and product validation.

## Figures and Tables

**Table 1.** Mode Shape and ODS comparative (adapted from Schwarz *et al* [3]).

**Mode Shape/ODS**	**Frequency**	**Modes**	**Depend on Force/Load**	**Description**
Mode Shape	Natural	Linear	No	Relative motion of one DOF *vs.* another
ODS	Any	Non linear	Yes	Actual motion of one DOF *vs.* another

**Table 2.** ODS Measurement setup.

**Parameter**	**Value**	**Value**
Channel	1	2
Frequency Range	0–500 Hz	0–500 Hz
Number of spectral lines	800	800
Resolution	1.6 Hz	1.6 Hz
Number of Averages	25	25
Window	Hannig	Hannig
Sensor Sensitivity	Reference: 1 pc/m^2^	Response: 0.114 pc/m^2^

**Table 3.** Factors of computational parametric analysis.

**Factor**	**Levels**	**Description**
Polyurethane Core Thickness	2	Core composite thickness: 6 mm–12 mm
Polyurethane Type	2	Density of Polyurethane Foam: a: Low; b: High
Outer Layers Type	3	Outer layers Stiffness: Low-Medium-High
Adhesive Type	2	Density of Adhesive: aa: Low; bb: High

**Table 4.** Naked Metal Sheet FEM *vs.* ODS Frequencies.

**Mode**	**FEM (Hz)**	**ODS (Hz)**	**Error (%)**	**Shape**
1	50.8	50.6	0.4	(1, 1)
2	73.7	73.8	0.1	(2, 1)
3	113.2	114	0.7	(3, 1)
4	127.5	123	3.5	(1, 2)
5	148.8	148	0.5	(2, 2)
6	168.3	168	0.2	(4, 1)
7	185.6	186	0.2	(2, 3)
8	243.4	233	2.2	(1, 3)
9	238.9	238	0.4	(5, 1)

**Table 5.** Naked Metal Sheet plus Lightweight Multilayer Composite FEM *vs.* Naked Metal Sheet plus Lightweight Multilayer Composite OSD Frequencies.

**Mode**	**FEM (HZ)**	**ODS (HZ)**	**Error (%)**	**Shape**
1	85.9	85.6	0.37	(1, 1)
2	120.0	118.1	1.58	(2, 1)
3	165.9	156.9	5.75	(3, 1)
4	180.5	166.3	8.54	(1, 2)
5	205.0	189.4	8.26	(2, 2)
6	220.4	221.9	0.66	(4, 1)
7	243.3	238.1	2.16	(2, 3)
8	285.1	288.1	1.06	(1, 3)
9	301.6	301.3	0.12	(5, 1)

**Table 6.** 1st Mode: analysis of variance.

**1st Mode**		**SS**	**DF**	**RMS**	**F-Ratio**	***P*-Value**	**Contribution (%)**
Main factors	A: Thickness	331.5270	1	331.5270	6938.9300	<0.0001	22.98
	B: Foam	1.1267	1	1.1267	23.5800	0.0009	0.08
	C: Outer layers	1092.5400	2	546.2720	11433.5900	<0.0001	75.74
	D: Adhesive	0.1067	1	0.1067	2.2300	0.1693	0.01

Interactions	AB	0.6017	1	0.6017	12.5900	0.0062	0.04
	AC	16.2233	2	8.1117	169.7800	<0.0001	1.12
	AD	0.0017	1	0.0017	0.0300	0.8560	0.00
	BC	0.0033	2	0.0017	0.0300	0.9658	0.00
	BD	0.0017	1	0.0017	0.0300	0.8560	0.00
	CD	0.0133	2	0.0067	0.1400	0.8716	0.00

Error		0.4300	9	0.0478	-	-	0.03

Corrected Total		1442.5800	23	-	-	-	-

**Table 7.** 2nd Mode: Analysis of Variance.

**2nd Mode**		**SS**	**DF**	**RMS**	**F-Ratio**	***P*-Value**	**Contribution (%)**
Main factors	A: Thickness	495.0420	1	495.0420	24,525.000	<0.0001	22.03
	B: Foam	0.0267	1	0.0267	1.3200	0.2800	0.00
	C: Outer layers	1738.0400	2	869.0200	43,052,390	<0.0001	77.35
	D: Adhesive	0.0600	1	0.0600	2.9700	0.1188	0.00

Interactions	AB	0.0017	1	0.0017	0.0800	0.7804	0.00
	AC	13.2708	2	6.6354	328.7300	<0.0001	0.59
	AD	0.0817	1	0.0817	4.0500	0.0752	0.00
	BC	0.1258	2	0.0629	3.1200	0.0936	0.01
	BD	0.0600	1	0.0600	2.9700	0.1188	0.00
	CD	0.1425	2	0.0713	3.5300	0,.738	0.01

Error		0.1817	9	0.0202	-	-	0.01

Corrected Total		2247.0300	23	-	-	-	-

**Table 8.** 3rd Mode: Analysis of Variance.

**3rd Mode**		**SS**	**DF**	**RMS**	**F-Ratio**	***P*-Value**	**Contribution (%)**
Main Factors	A: Thickness	624.2400	1	624.2400	1074.2200	<0.0001	12.33
	B: Foam	1.1267	1	1.1267	1.9400	0.1972	0.02
	C: Outer Layers	4405.9900	2	2203.0000	3791.0100	<0.0001	87.01
	D: Adhesive	0.0067	1	0.0067	0.0100	0.9171	0.00

Interactions	AB	3.6817	1	3.6817	6.3400	0.0329	0.07
	AC	14.1175	2	7.0588	12.1500	0.0028	0.28
	AD	0.0417	1	0.0417	0.0700	0.7949	0.00
	BC	9.2808	2	4.6404	7.9900	0.0101	0.18
	BD	0.0417	1	0.0417	0.0700	0.7949	0.00
	CD	0.0408	2	0.0204	0.0400	0.9656	0.00

Error		5.2300	9	0.5811	-	-	0.10

Corrected Total		5063.8000	23	-	-	-	-
